# Co-expression of fibrotic genes in inflammatory bowel disease; A localized event?

**DOI:** 10.3389/fimmu.2022.1058237

**Published:** 2022-12-23

**Authors:** Nikolas Dovrolis, Eirini Filidou, Gesthimani Tarapatzi, Georgios Kokkotis, Michail Spathakis, Leonidas Kandilogiannakis, Ioannis Drygiannakis, Vassilis Valatas, Konstantinos Arvanitidis, Ioannis Karakasiliotis, Stergios Vradelis, Vangelis G. Manolopoulos, Vasilis Paspaliaris, Giorgos Bamias, George Kolios

**Affiliations:** ^1^ Laboratory of Pharmacology, Department of Medicine, Democritus University of Thrace, Alexandroupolis, Greece; ^2^ Laboratory of Biology, Department of Medicine, Democritus University of Thrace, Alexandroupolis, Greece; ^3^ Individualised Medicine & Pharmacological Research Solutions Center (IMPReS), Alexandroupolis, Greece; ^4^ Gastrointestinal (GI) Unit, 3 Department of Internal Medicine, Sotiria Hospital, National and Kapodistrian University of Athens, Athens, Greece; ^5^ Gastroenterology and Hepatology Research Laboratory, Medical School, University of Crete, Heraklion, Greece; ^6^ Second Department of Internal Medicine, University Hospital of Alexandroupolis, Democritus University of Thrace, Alexandroupolis, Greece; ^7^ Tithon Biotech Inc., San Diego, CA, United States

**Keywords:** fibrosis, IBD, co-expression, tissue localization, transcriptomics

## Abstract

**Introduction:**

Extracellular matrix turnover, a ubiquitous dynamic biological process, can be diverted to fibrosis. The latter can affect the intestine as a serious complication of Inflammatory Bowel Diseases (IBD) and is resistant to current pharmacological interventions. It embosses the need for out-of-the-box approaches to identify and target molecular mechanisms of fibrosis.

**Methods and results:**

In this study, a novel mRNA sequencing dataset of 22 pairs of intestinal biopsies from the terminal ileum (TI) and the sigmoid of 7 patients with Crohn’s disease, 6 with ulcerative colitis and 9 control individuals (CI) served as a validation cohort of a core fibrotic transcriptomic signature (FIBSig), This signature, which was identified in publicly available data (839 samples from patients and healthy individuals) of 5 fibrotic disorders affecting different organs (GI tract, lung, skin, liver, kidney), encompasses 241 genes and the functional pathways which derive from their interactome. These genes were used in further bioinformatics co-expression analyses to elucidate the site-specific molecular background of intestinal fibrosis highlighting their involvement, particularly in the terminal ileum. We also confirmed different transcriptomic profiles of the sigmoid and terminal ileum in our validation cohort. Combining the results of these analyses we highlight 21 core hub genes within a larger single co-expression module, highly enriched in the terminal ileum of CD patients. Further pathway analysis revealed known and novel inflammation-regulated, fibrogenic pathways operating in the TI, such as IL-13 signaling and pyroptosis, respectively.

**Discussion:**

These findings provide a rationale for the increased incidence of fibrosis at the terminal ileum of CD patients and highlight operating pathways in intestinal fibrosis for future evaluation with mechanistic and translational studies.

## 1 Introduction

Intestinal fibrosis is a feature of complicated Inflammatory Bowel Diseases (IBD), with Crohn’s Disease (CD) significantly more affected compared to Ulcerative Colitis (UC). This can be attributed to fibrosis in CD being a transmural process, while in UC, limited to the lamina propria (1). Nonetheless, fibrosis is a serious manifestation for both diseases, as it may lead to motility disorders and intestinal obstruction. There is no medical treatment for intestinal fibrosis yet (2).

In physiology, a trauma on mucosal surface induces inflammation. As inflammation fades, mesenchymal cells, the main extracellular matrix (ECM) producers, are recruited to promote wound healing (3). In IBD pathophysiology, the dynamic balance between secreted extracellular matrix (ECM) components and enzymes dissolving it, is disturbed towards fibrogenesis (4).

In this process, various pro-fibrotic signaling pathways are involved, all of them leading to either the upregulation of secretion of ECM components, such as distinct types of collagens and fibronectin, or the imbalanced expression between metalloproteinases (MMPs), which degrade ECM, and tissue inhibitors of metalloproteinases (TIMPs), which counteract MMPs’ activity (5). The most well-known profibrotic signaling pathway is that of *TGF-β*, acting either through canonical signaling that involves the activation of Smads, or through two Smad-independent signaling pathways: mitogen-activated protein kinases (*MAPK*) and phosphatidylinositol 3-kinases (*PI3K*). Other significant pathways include Wnt/β-Catenin, Sonic hedgehog (*Shh*), Notch and Integrin-linked kinases, all of which lead to upregulated expression of ECM and, ultimately, result in fibrogenesis (5).

Apart from IBD, fibrosis is a common complication of various diseases, such as Idiopathic Pulmonary Fibrosis (IPF) (6), Chronic Kidney Disease (CKD) (7), Systemic Sclerosis (SSc) (8) and Chronic Liver Diseases (CLD) (9) and in all cases, a successful anti-fibrotic treatment is yet to be found. Regarding intestinal fibrosis, it is most commonly symptomatic in CD rather than in UC, and in some cases, its occurrence may lead to intestinal strictures, which are amenable only to excision (2). Around 10% of patients with CD develop a stricturing phenotype (10), out of which 40-70% will require surgical intervention at least once, often due to stricture (11). Although strictures can occur in any part of the gastrointestinal tract, the most commonly affected segment is the small bowel and more specifically, the terminal ileum (12). Around 40-55% of *de novo* strictures occur in the terminal ileum, while a lower prevalence has been reported for other parts of the gastrointestinal tract (13). Despite clinical awareness, there are still a few studies attempting to shed light to implicated pathogenetic mechanisms in the terminal ileum, where stenoses will be most likely to be symptomatic (14–22).

The first aim of this study was to demonstrate an *in silico* methodology for the identification of genes and pathways involved in profibrotic mechanisms common between different fibrotic disorders of different organs as well as their site-specific occurrence. For this purpose, publicly available data for 5 such diseases were used to assemble a core fibrotic transcriptomic signature (FIBSig). The second aim was to validate FIBSig with wet-lab experiments. In more detail, we carried out mRNA sequencing of paired intestinal biopsies from the sigmoid and the terminal ileum of CD, UC, and control individuals (CI). We expanded on pathways involved and focused on ileum-specific ones, as this may reflect modalities of greater clinical impact and of interest in therapeutics.

## 2 Materials and methods

### 2.1 Public data

#### 2.1.1 Common fibrotic signature *via* differential gene expression

To identify genes which are commonly dysregulated between CD, IPF, CKD, SSc and CLD, we used publicly available gene expression data from NCBI’s Gene Expression Omnibus (GEO) (23). In total 9 CD [GSE3365 (24), GSE6731 (25), GSE9686 (26), GSE16879 (27), GSE20881 (28), GSE59071 (29), GSE75214 (30), GSE94648 (31), GSE97012 (32)], 2 IPF [GSE93606 (33), GSE110147 (34)], 1 CKD [GSE66494 (35)], 1 CLD [GSE17548 (36)] and 1 SSc [GSE76807 (37)] datasets were retrieved from GEO. All datasets fulfill the following criteria: only human subjects, each dataset with both patients and controls and only created by microarray experiments. Only samples of interest, preferably prior to therapeutic interventions, were utilized making a total of n=839.

Differential gene expression of patients versus controls was calculated with the GEO2R tool in each dataset, to decrease experimental bias. Genes of perturbed expression, statistically significant by the linear models for microarray data (limma) method (38) at the p < 0.05 level, were extracted for each individual dataset.

To identify commonalities among these gene lists, a multiset intersection approach was adopted using the R package SuperExactTest v1.1.0 (39). Initially, the 9 gene lists derived from CD datasets were intersected among themselves. The gene lists from the other 4 fibrotic disorders were separately intersected. Constructing two separate gene lists (CD, the other 4 fibrotic diseases) aimed to provide higher precision while maintaining a broader point of view on common differentially expressed genes. The first list consisted of genes of at least 7 out of 9 CD gene lists, which includes C(9,7)=36 combinations, C(9,8)=9 combinations and C(9,9)=1 combination, making a total of 46 combinations (GeneSet1). Similarly for the rest (5) gene lists of the other 4 fibrotic conditions the combined gene set was created by all combinations of at least 4 out of 5 gene lists, which includes C(5,4)=5 combinations and C(5,5)=1 combination, making a total 6 combinations (GeneSet2). This approach allows for all datasets to be utilized without losing information which does not fit on strict comparisons. The last step was to intersect GeneSet1 and GeneSet2 and aggregate all the common genes to a gene list, hereafter called fibrotic signature (FIBSig).

#### 2.1.2 Literature-based tissue-specific co-expression analysis

To study how the FIBSig genes are co-expressed in the sigmoid and the terminal ileum we used them as input for the gene co-expression network analysis module of the online platform NetworkAnalyst v3.0 (40). This module is based on data from the iNetModels (41) database and provides users with information on how specific genes are co-expressed in various tissues. Co-expression analysis identifies clusters of genes (functional gene modules) which follow similar expression patterns across samples, identifying associations with specific factors (42).

#### 2.1.3 Pathway enrichment analysis

All functional analyses of this work were performed in R using the clusterProfiler v4.0.5 (43) package using knowledge from the following databases: Reactome (44), Gene Ontology:Biological Process (GO:BP) (45), Gene Ontology: Molecular Function (GO:MF) (45) and Kyoto Encyclopedia of Genes and Genomes (KEGG) (46). Enrichment p-values were adjusted using the Benjamini-Hochberg method. Based on the literature these databases provide some overlap but also unique insights into gene contribution on biological functions (47, 48).

### 2.2 RNA-SEQ from collected intestinal biopsies

#### 2.2.1 Patients

Paired intestinal biopsies from the sigmoid and the terminal ileum were obtained with endoscopy from 9 individuals without autoimmune disease, malignancy or acute infection, who underwent screening colonoscopy and had no abnormal findings (control individuals- CI), 7 patients with CD and 6 with UC. Endoscopies were performed at the Endoscopy Department, University Hospital of Alexandroupolis, Greece. The local Research Ethics Committee approved this study (Protocol Number: Θ9/Δ.Σ37/21.12.2018), and patients gave their informed written consent prior to participation. Upon retrieval, biopsies were immediately submerged in RNAlater (Sigma-Aldrich, St. Louis, Missouri, United States) and stored at -80^o^C until further processing. **Table 1** summarizes the metadata of the 44 samples.

**Table 1:** Demographic characteristics of included samples.

SAMPLE NAME	PATIENT ID	TISSUE	CONDITION	SEX	AGE
1HI	B1_B2	ILEAL	HI	F	61
2HI		SIGMOID			
13UC	B13_B14	ILEAL	UC	F	52
14UC		SIGMOID			
15UC	B15_B16	ILEAL	UC	F	53
16UC		SIGMOID			
17UC	B17_B18	ILEAL	UC	M	27
18UC		SIGMOID			
19UC	B19_B20	ILEAL	UC	M	33
20UC		SIGMOID			
21UC	B21_22	ILEAL	UC	M	67
22UC		SIGMOID			
23UC	B23_B24	ILEAL	UC	F	61
24UC		SIGMOID			
3CD	B3_B4	ILEAL	CD	F	52
4CD		SIGMOID			
31CD	B31_B32	ILEAL	CD	F	29
32CD		SIGMOID			
33CD	B33_B34	ILEAL	CD	M	51
34CD		SIGMOID			
35CD	B35_B36	ILEAL	CD	F	60
36CD		SIGMOID			
41HI	B41_B42	ILEAL	HI	F	62
42HI		SIGMOID			
43HI	B43_B44	ILEAL	HI	F	54
44HI		SIGMOID			
45HI	B45_B46	ILEAL	HI	M	57
46HI		SIGMOID			
47HI	B47_B48	ILEAL	HI	M	69
48HI		SIGMOID			
49HI	B49_B50	ILEAL	HI	M	58
50HI		SIGMOID			
5CD	B5_B6	ILEAL	CD	M	44
6CD		SIGMOID			
51HI	B51_B52	ILEAL	HI	F	72
52HI		SIGMOID			
53HI	B53_B54	ILEAL	HI	M	74
54HI		SIGMOID			
55HI	B55_B56	ILEAL	HI	M	55
56HI		SIGMOID			
7CD	B7_B8	ILEAL	CD	M	28
8CD		SIGMOID			
10CD	B9_B10	SIGMOID	CD	F	35
9CD		ILEAL			

#### 2.2.2 Total RNA extraction and purification

Total RNA from tissue biopsies was extracted and purified from DNA traces using the Nucleospin RNA Plus XS kit (MACHEREY-NAGEL, Düren, Germany) according to the manufacturer’s instructions. Briefly, tissues were first lysed and homogenized and DNA was removed by passing the lysate through DNA-removal columns. The purified lysate was then loaded onto RNA-extraction columns, washed 3 times and finally total RNA was eluted using Rnase-free H_2_O. The concentration and purity of total RNA was measured in a Q5000 UV-Vis spectrophotometer (Quawell, San Jose, California, United States).

#### 2.2.3 RNA sequencing, alignment and PCA

Next Generation Sequencing (NGS) libraries from the total RNA samples were prepared using a QuantSeq 3′ mRNA-Seq Library Prep Kit FWD according to the manufacturer’s instructions. Sequencing was carried out on a IonTorrent S5 sequencer (Thermo Fisher Scientific Inc,Waltham, Massachusetts, USA). Output averaged ~3-4 million quality-controlled reads per sample with a median read length of 140bp.

Alignment of sequences was performed using Salmon v.1.6.0 (49) on the GRCh38 Human Transcriptome reference. Salmon’s output was imported into R using tximport v1.20.0 (50) as a DESEQ2 v1.32.0 (51) object. Principal Component Analysis (PCA) was performed on the transformed (normTransform function) DESEQ2 object using the plotPCA function.

#### 2.2.4 RNA-seq Co-expression Gene set enrichment analysis

The normalized counts of the FIBSig genes only were used as input to CEMiTool v1.16.0 (52) in R to perform automatic analysis and discovery of co-expression modules across our specified conditions (CD, UC, and HI) and intestinal site (sigmoid and terminal ileum). In addition, a protein-protein interaction (PPI) network constructed via STRING-db (53), by the genes identified by CEMiTool as co-expressed, was fed back as additional input to CEMiTool which, in turn, highlighted several hub genes involved via network centrality analysis.

## 3 Results

### 3.1 *In silico* data

#### 3.1.1 Fibrotic signature genes

**Figure 1:**
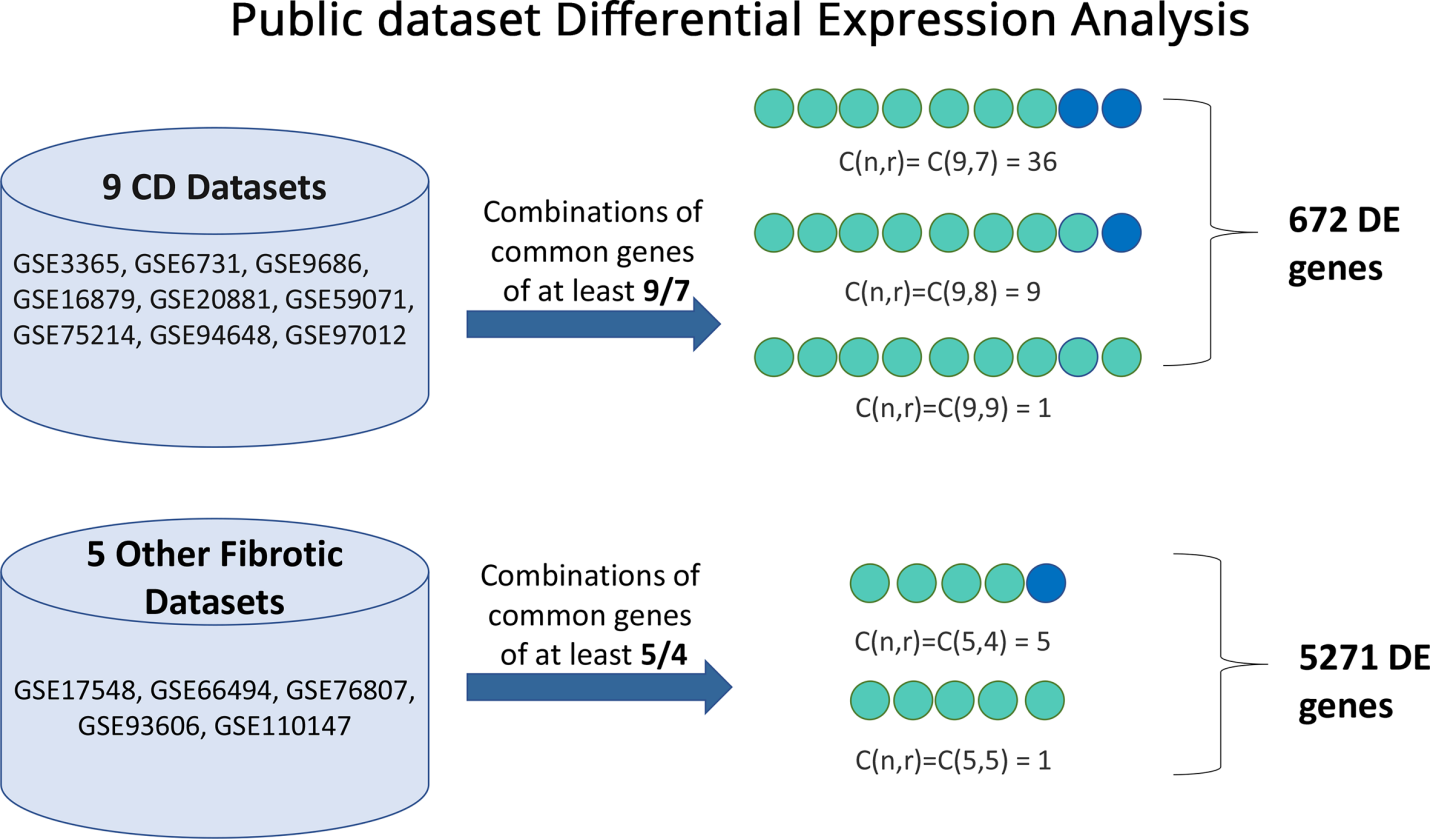
Visual representation of the differential expression analysis pipeline employed to identify fibrosis-related genes from public datasets of 5 fibrotic disorders (Crohn’s Disease, Idiopathic Pulmonary Fibrosis, Chronic Kidney Disease, Systemic Sclerosis and Chronic Liver Disease) using the GEO2R online tool. In total, 46 combinations of the Crohn’s Disease datasets yielded 672 differentially expressed genes and 6 combinations of the datasets from other fibrotic disorder highlighted 5271 differentially expressed genes. Multiset comparisons of combinations were conducted using the SuperExactTest R package.

**Figure 2:**
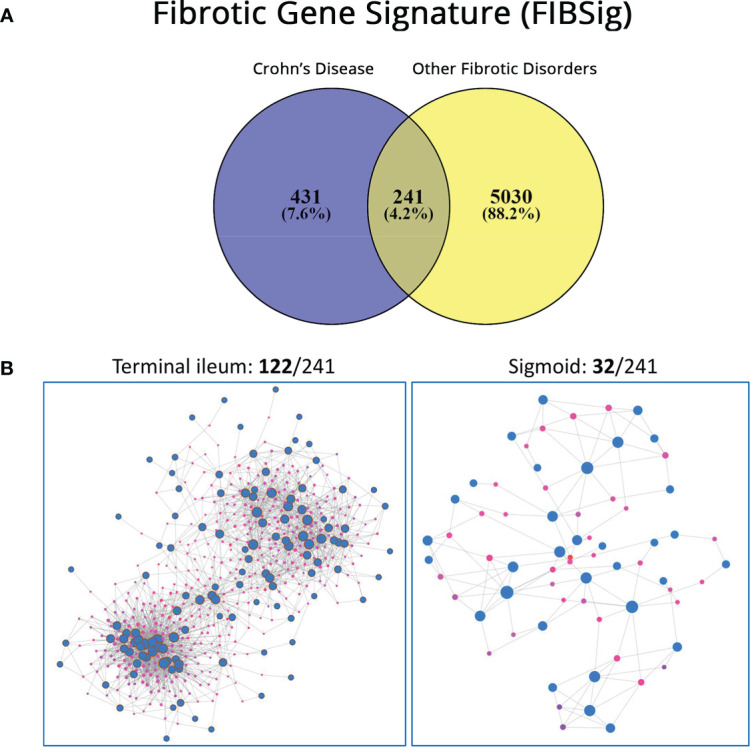
**(A)** Venn diagram reporting on genes expressed in Crohn’s Disease, in other fibrotic diseases and their intersection (FIBSig). **(B)** Tissue-specific expression with the NetworkAnalyst online platform and information form the iNetModels database; significantly more FIBSig genes were co-expressed in the terminal ileum versus the sigmoid.

We first utilized public data to identify and explore a common signature in various fibrotic disorders. **Figure 1** depicts the methodology used and the resulting gene sets arising from the multiset intersections of differentially expressed genes (DEG). The complete DEG sets from each individual analysis can be found in the **Supplementary File**. Seven genes (*CXCL1, ICAM1, PHLPP2, ZKSCAN1, ATP9A, NCF4, CACNA2D1*) were common in all CD datasets. In total, gene expression of 672 genes was found commonly perturbed in the CD datasets and 5271 genes in the other 4 disorders. Their intersection, hereafter referred to as fibrotic signature (FIBSig), includes 241 genes (**Figure 2A**; **Table 2**). Many of these genes were involved in immunology and inflammation pathways, which are upstream of fibrosis end-products. This was promising and prompted us to further investigation of their synergies and contribution to pathophysiology mechanisms.

**Table 2:** Transcriptomic signature of 241 genes commonly dysregulated in all fibrotic disorders under study.

*ICAM1*	*VCP*	*SLC16A5*	*TRAM2*	*HSP90B1*	*OGFR*	*AP3D1*	*CDKN2B*
*ZKSCAN1*	*TGM2*	*SSR1*	*RAB17*	*RPS6KA2*	*HNRNPU*	*PDS5A*	*SPRYD7*
*ATP9A*	*RECQL*	*IRF1*	*ABCA5*	*TPK1*	*SMARCA2*	*CCND1*	*SASH1*
*NAMPT*	*IL1RAP*	*CEBPB*	*CXCL5*	*AVIL*	*SLC9A6*	*ROCK2*	*RAMP3*
*S100A11*	*STAT1*	*PSME1*	*TPM4*	*HADHA*	*CDC6*	*UBXN4*	*OCRL*
*SUPT4H1*	*PTPN21*	*HDLBP*	*LETM1*	*MMP1*	*MELTF*	*TAOK3*	*SLC4A7*
*ASPH*	*PPM1A*	*AQP9*	*CLEC7A*	*PDLIM2*	*TCF12*	*STRN*	*TNC*
*ACSL1*	*GM2A*	*PLCB1*	*FBXW7*	*TMEM38B*	*HNRNPH3*	*GATAD1*	*SS18L1*
*DRAM1*	*TMEM41B*	*EIF4EBP2*	*CCL2*	*HSPA13*	*CALM3*	*MUT*	*RABGAP1*
*VNN1*	*ATP2C1*	*IL7R*	*TRIM22*	*NUCB2*	*RAP1A*	*RMND5A*	*CCNT2*
*C1QA*	*SYNGR2*	*PYHIN1*	*SCD*	*SERPING1*	*ENDOD1*	*DST*	*EIF4G3*
*HNF4A*	*CFB*	*TRABD*	*TMEM184B*	*ARMCX3*	*QKI*	*KIAA1109*	*DCAF4*
*SOCS3*	*CADM1*	*PATJ*	*IFITM2*	*HLA-DQB1*	*TNFRSF1B*	*NDUFA5*	*CALD1*
*XPO1*	*SNX13*	*RANBP9*	*TUBB6*	*NOL7*	*LRPPRC*	*RYBP*	*DYRK2*
*MYCBP2*	*ARF6*	*PFKFB3*	*MBNL3*	*SMC3*	*LTN1*	*SOCS1*	*NDUFAF3*
*PRRC2C*	*DEF8*	*MLLT10*	*ADAM9*	*PHACTR2*	*PAPSS2*	*ARHGEF10*	*SORL1*
*NUP210*	*GPCPD1*	*BMPR1A*	*BAG2*	*PGLYRP1*	*MEST*	*DLD*	*SPAG9*
*ATRX*	*DDAH2*	*NEDD4L*	*HEXIM1*	*KAT6B*	*RRAS2*	*CHMP2B*	*CDC5L*
*LIMK1*	*CASP1*	*TUSC2*	*SLC7A11*	*CD58*	*ZNF292*	*NRP2*	*SERHL2*
*NDUFA6*	*FBXL20*	*SYF2*	*BHLHE40*	*ATM*	*ZNF43*	*PPP6C*	*FLT3*
*ETS1*	*MRPS25*	*SETX*	*OSBPL3*	*PHF3*	*KYNU*	*CREBL2*	*TFPI2*
*ADAMTS2*	*CLDN1*	*PHF21A*	*RRM2*	*DLG1*	*RHOQ*	*API5*	*PLA2R1*
*SPON1*	*MIEN1*	*STX11*	*IGFBP7*	*FOXN3*	*SERBP1*	*GMFB*	*KIAA0930*
*PEX1*	*CASP4*	*CBFA2T2*	*COL4A1*	*REL*	*OAZ1*	*SEMA3F*	*BRD3*
*ZEB2*	*DAAM1*	*GIGYF2*	*RARRES3*	*ITGA6*	*SCAMP3*	*CD3E*	*TNPO2*
*RNF14*	*ARHGAP1*	*ANXA3*	*HIF1A*	*PRKD3*	*SBF1*	*SKIL*	*MECOM*
*APPBP2*	*ESR1*	*PHTF1*	*ARMC8*	*ARHGAP5*	*BCAT1*	*RNF6*	*KBTBD11*
*PAFAH1B1*	*COBLL1*	*EDN2*	*THEMIS2*	*MGAT5*	*MMP14*	*GSR*	*CDC14B*
*PAX8*	*ATP6V1C1*	*TAF4*	*THRA*	*FGFR1*	*NDUFB8*	*FEZ2*	*UBE4A*
*PHLDA1*	*ZNF81*	*TRIB1*	*PGM3*	*NIPAL3*	*NFYC*	*CNBP*	*OLFM1*
*MTDH*							

This gene set was named FIBSig.

#### 3.1.2 Intestinal site-specific genes

Keeping in mind that ileal fibrosis is far more common and debilitating than colonic fibrosis, we focused on finding if FIBSig was involved. When FIBSig was used as input to NetworkAnalyst’s co-expression analysis module, it revealed that 122 of these 241 genes were co-expressed in the terminal ileum and 32 of the 241 genes in the sigmoid (**Figure 2B**), revealing a higher involvement of the FIBSig in the terminal ileum. 20 genes (*ARHGEF10, QKI, ETS1, IFITM2, GPCPD1, ZEB2, OSBPL3, SOCS3, CCL2, CADM1, DDAH2, CEBPB, ACSL1, NAMPT, TFPI2, HIF1A, ADAMTS2, SCD, TRAM2, PFKFB3*) were common in both tissues, defining a shared co-expression module. The complete gene sets can be found in the **Supplementary File**.

#### 3.1.3 Biological pathway analysis of FIBSig

Functional analysis of the FIBSig genes was conducted using literature and experimental information provided by the databases described in the methodology.

**Figure 3:**
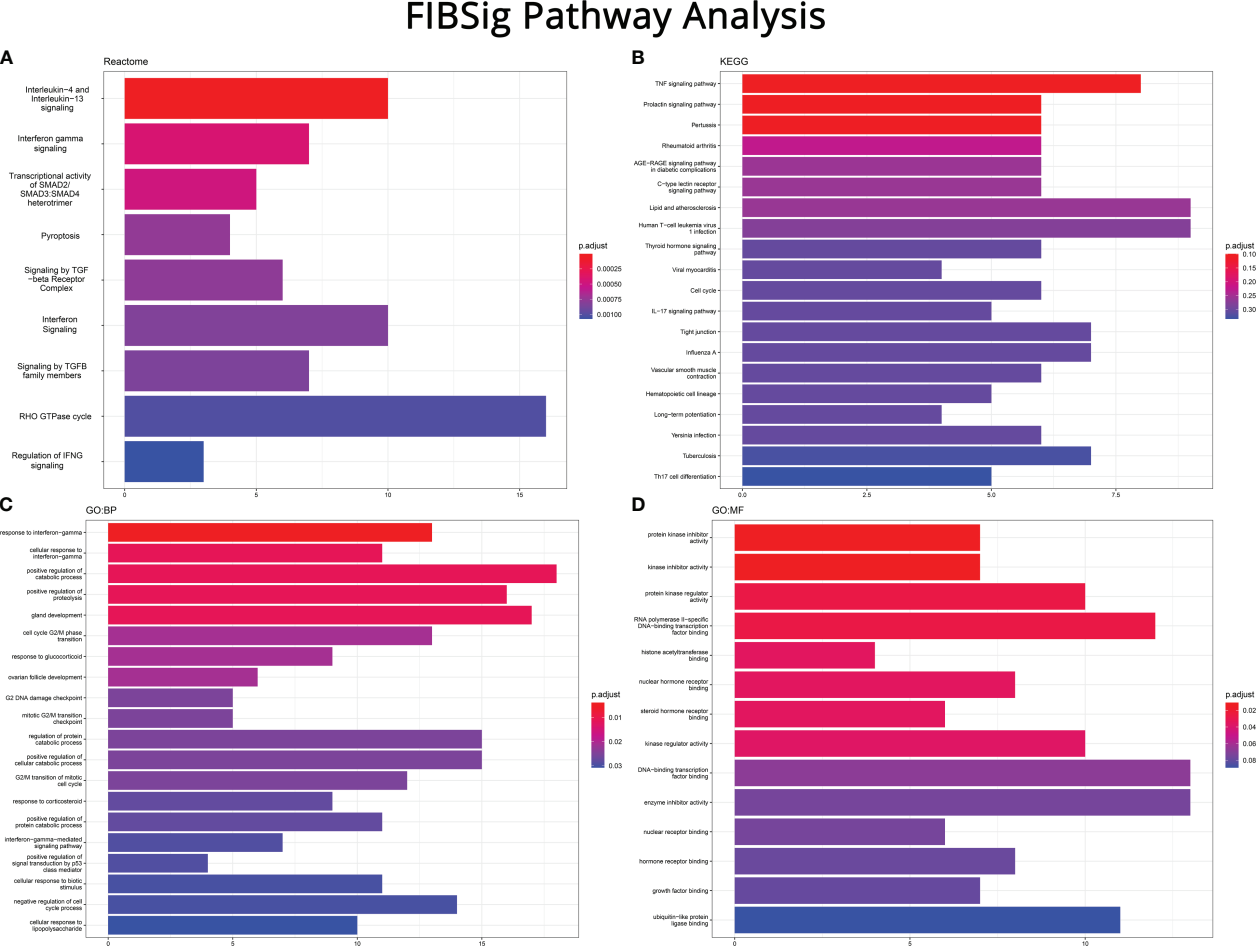
Pathway enrichment analysis on the FIBSig genes (performed with R and the clusterProfiler package) using **(A)** Reactome, **(B)** Kyoto Encyclopedia of Genes and Genomes (KEGG), **(C)** Gene Ontology: Biological Process (GO:BP) and **(D)** Gene Ontology: Molecular Function (GO:MF). Top to bottom: most to least FIBSIg genes as a ratio of the pathway’s total number of genes.

Reactome revealed their high involvement in significant inflammatory and fibrogenic pathways, like modifying *TGF-β, SMAD* 2/3/4 heterotrimer and *RHO GTPase.* Enrichment of cytokine pathways, including interferons and interleukins verified that immune signaling precedes and parallels fibrosis. The combined pathway of T helper (Th)2 interleukins (IL-) 4 and 13, along with interferon (*IFN*) gamma further supported their role in fibrogenesis. Pyroptosis, a type of cell death caused by inflammation, was also identified by Reactome, indicating that cell death is related to fibrosis. A complete list of involved pathways can be reviewed in **Figure 3A**.

By design, KEGG features pathways which point to alternative disorders of similar molecular mechanisms. As seen in **Figure 3B**, results such as pertussis, rheumatoid arthritis, atherosclerosis, leukemia, viral myocarditis, influenza A, Yersinia infection and tuberculosis can be viewed in regard to mechanisms controlling their onset and progression. Several other pathways, like prolactin signaling and hematopoietic cell lineage, appear to be off-target results or of unknown importance. However, the fact that *TNF*, *IL-17* and T helper (Th)17 differentiation pathways were enriched, also emphasized the crucial role of innate and adaptive immunity in the pathogenesis of fibrosis. Furthermore, involvement of the tight junction pathway stressed the importance of the integrity of the epithelial barrier to prevent profibrotic signaling in the lamina propria.

Results from the two Gene Ontology (**Figures 3C, D**) databases further broke down the grouped pathways of the previous analyses into more specific biological processes. For instance, *IFN* signaling was broken down to IFN-γ, cellular response to *IFN-γ* and *IFN-γ*-mediated signaling pathways. Similarly, the cell cycle pathway of KEGG was further divided into the G2/M phase transition, G2 DNA damage checkpoint, mitotic G2/M transition checkpoint, G2/M transition of mitotic cell cycle, positive regulation of signal transduction by p53 and negative regulation of cell cycle process pathways. In addition, the results of GO:MF brought up to the foreground that enzymes and receptors are also involved in fibrosis.

### 3.2 Validation

#### 3.2.1 mRNA sequencing

**Figure 4:**
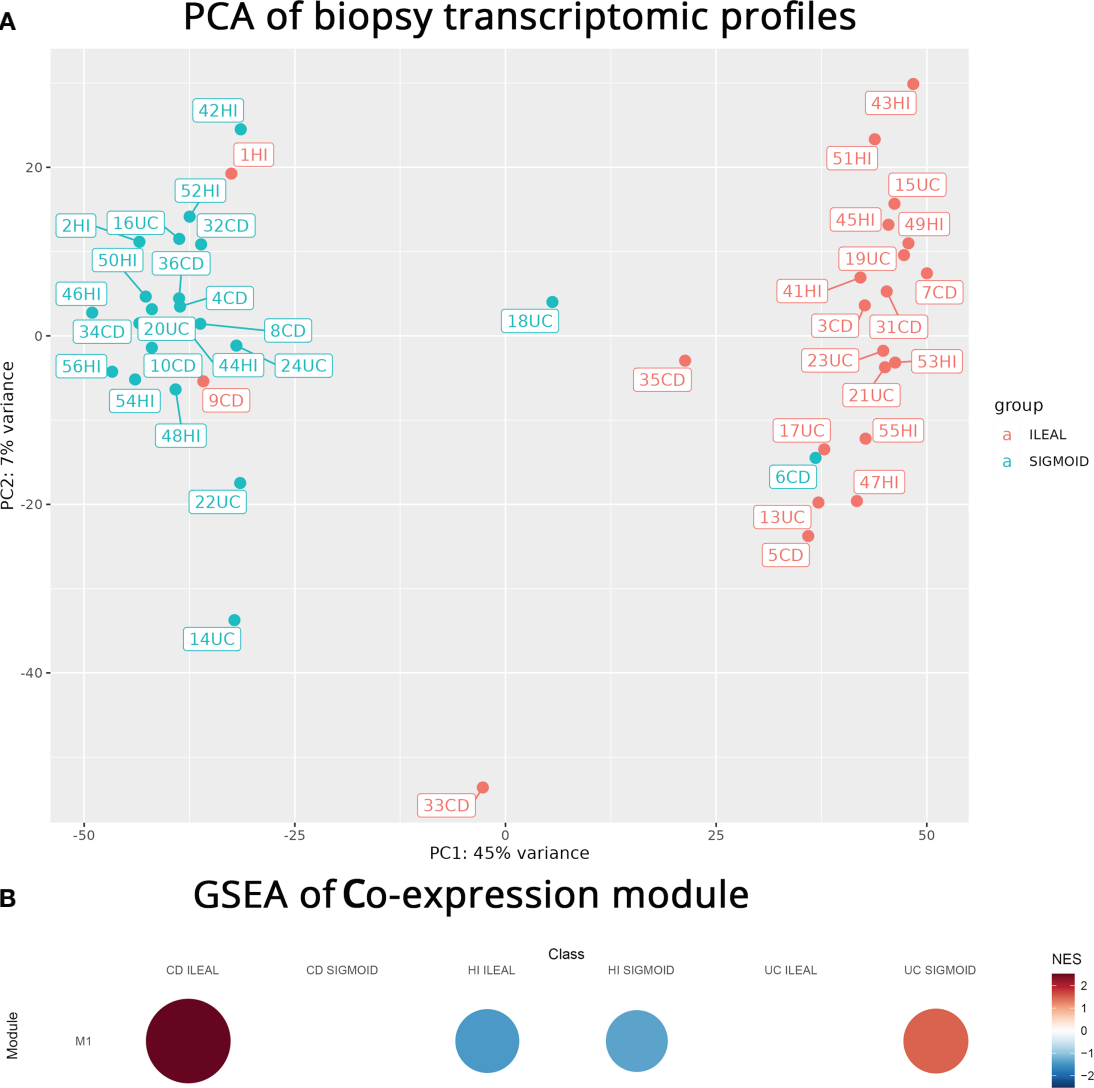
**(A)** PCA plot of our mRNA sequencing data colored according to biopsy location. Biopsy location explains a very large part of the variance. **(B)** Gene set enrichment analysis (GSEA) plot, generated with CEMiTool, showing the correlation of the co-expression module to specific disease and intestinal segment combinations. Red and blue colors denote over- or under-expression, respectively. Size depicts the strength of the correlations.

To generate independent data for validation of the aforementioned *in silico* findings, we performed mRNA sequencing on our own paired (terminal ileum, sigmoid) intestinal CD, UC, CI biopsies. PCA analysis (**Figure 4A**) of this sequencing effort pointed to the intestinal segment of origin having the largest effect on transcriptomic profiles compared to other factors, namely disease. In other words, most samples were well separated by intestinal segment of origin with the first PCA axis explaining a surprisingly substantial portion (45%) of the variance. Distinct transcriptomic profiles by intestinal segment held promise of this separation being maintained after subsetting the genes involved to include only those related to profibrotic signaling.

#### 3.2.2 Localization

To test this hypothesis, we focused on genes differentially expressed both in our mRNA sequencing effort and in FIBSig. Co-expression analysis was performed on the read counts of the FIBSig genes in our samples and, indeed, provided a single strong module (M1) of co-expression. The M1 module contained 112 genes (**Table 3**) of the 241 used as input (FIBSig).

**Table 3:** Genes comprising the M1 co-expression module.

***ICAM1***	***ADAMTS2***	***CASP1***	***SETX***	***IFITM2***	***PGM3***	***TNFRSF1B***	***CDKN2B***
***ZKSCAN1***	*ZEB2*	*MRPS25*	*PHF21A*	*TUBB6*	*HSP90B1*	*LTN1*	*SPRYD7*
***ATP9A***	*PHLDA1*	*CASP4*	*STX11*	*MBNL3*	*RPS6KA2*	*PAPSS2*	*RAMP3*
***NAMPT***	*VCP*	*ESR1*	*ANXA3*	*ADAM9*	*MMP1*	*KYNU*	*OCRL*
***S100A11***	*TGM2*	*ATP6V1C1*	*PHTF1*	*BAG2*	*HSPA13*	*RHOQ*	*TNC*
***ACSL1***	*RECQL*	*SSR1*	*TRIB1*	*SLC7A11*	*NUCB2*	*BCAT1*	*RABGAP1*
***DRAM1***	*IL1RAP*	*IRF1*	*TRAM2*	*BHLHE40*	*SERPING1*	*MMP14*	*CALD1*
***HNF4A***	*STAT1*	*CEBPB*	*CXCL5*	*OSBPL3*	*ARMCX3*	*CCND1*	*DYRK2*
***SOCS3***	*PTPN21*	*PSME1*	*TPM4*	*RRM2*	*HLA-DQB1*	*RYBP*	*SORL1*
***MYCBP2***	*GM2A*	*AQP9*	*LETM1*	*IGFBP7*	*ATM*	*SOCS1*	*FLT3*
***PRRC2C***	*CFB*	*EIF4EBP2*	*CLEC7A*	*COL4A1*	*DLG1*	*NRP2*	*TFPI2*
***NUP210***	*CADM1*	*IL7R*	*FBXW7*	*HIF1A*	*PRKD3*	*CD3E*	*KIAA0930*
***ATRX***	*DEF8*	*PFKFB3*	*CCL2*	*THEMIS2*	*FGFR1*	*SKIL*	*OLFM1*
***ETS1***	*GPCPD1*	*NEDD4L*	*TRIM22*	*THRA*	*QKI*	*CNBP*	*MTDH*

To check if genes of M1 kept the ileal localization the originating pool (FIBSig) had, we proceeded to gene set enrichment analysis (GSEA) and found that the M1 module had a strong positive correlation with the terminal ileum samples of CD but was poorly correlated with the sigmoid in UC and was inversely correlated to all CI samples (**Figure 4B**). Positive Normalized Enrichment Score (NES) values in this case pointed to over-expressed genes that are co-expressed, while negative NES points to under-expression based on the way CEMiTool ranks the genes.

#### 3.2.3 Hub genes

**Figure 5:**
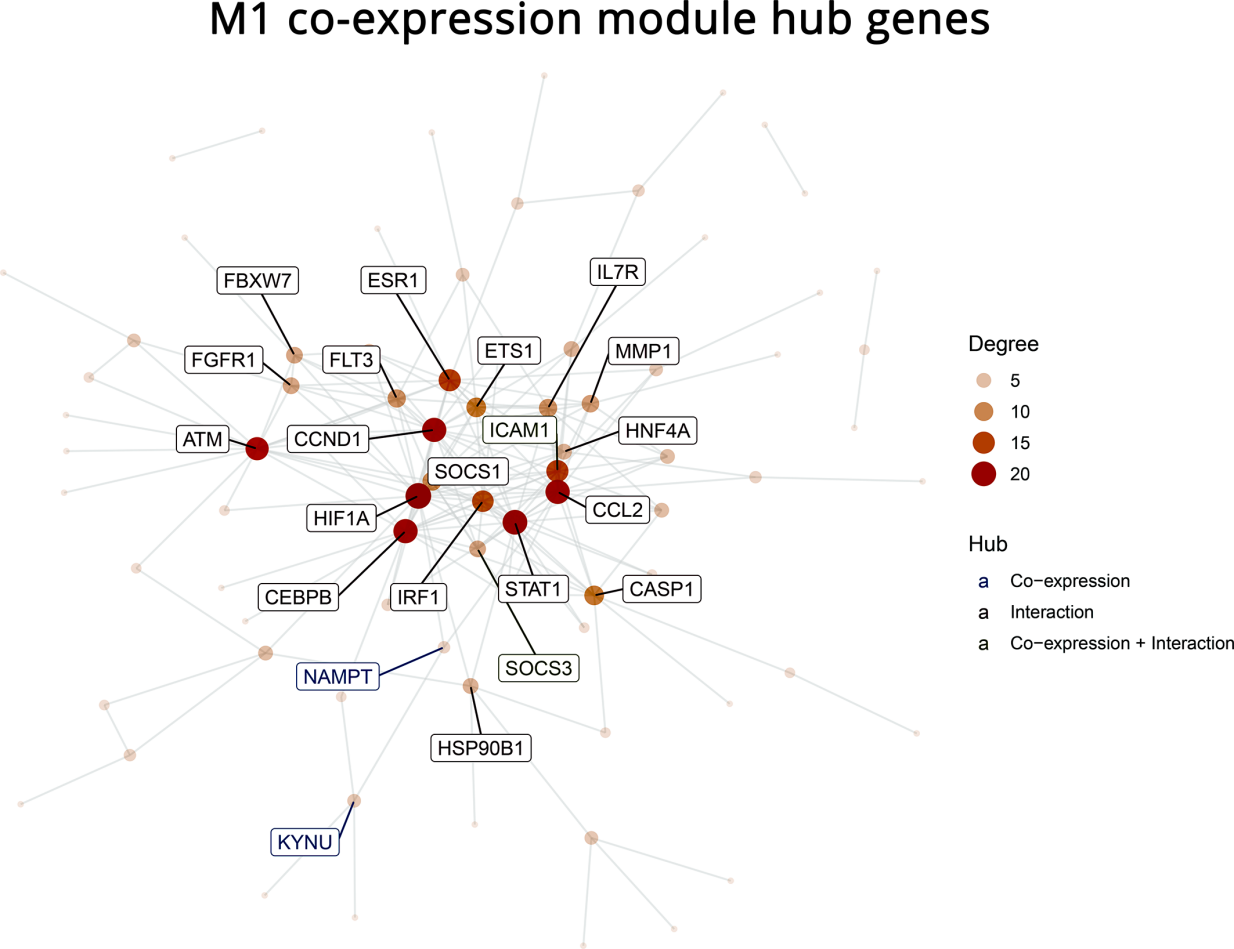
Protein-Protein Interaction (PPI) network of the 112 genes in the co-expression module M1. Twenty-one hub genes are labeled. The PPI was built with the STRING database based on known interactions between the provided genes. Hubs were defined as genes with a high degree centrality score, which signifies higher interconnectivity with other genes and importance for the stability of the network.

To narrow down the genes of the M1 module that hold more important roles than others in profibrotic pathways, a PPI network of the M1 module was constructed (full interactions provided in the **Supplementary File**). Twenty-one hub genes were identified as high degree nodes (*ATM, FGFR1, FBXW7, ESR1, CCND1, HIF1A, CEBPB, NAMPT, KYNU, IRF1, SOCS1, ICAM1, ETS1, IL7R, MMP1, HNF4A, CCL2, CASP1, STAT1, SOCS3, HSP90B1*). The importance of these hub genes in a biological network lies in their strong crosstalk with their gene neighbors via physical or signaling interactions (**Figure 5**).

#### 3.2.4 Profibrotic pathways

**Figure 6:**
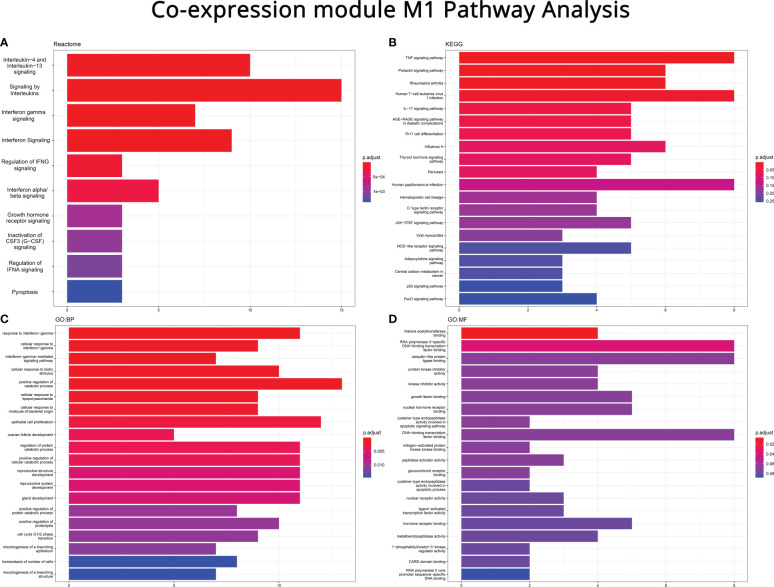
Pathway enrichment analysis on the M1 co-expression module genes (performed with R and the clusterProfiler package) using **(A)** Reactome, **(B)** Kyoto Encyclopedia of Genes and Genomes (KEGG), **(C)** Gene Ontology: Biological Process (GO:BP) and **(D)** Gene Ontology: Molecular Function (GO:MF). Top to bottom: most to least FIBSIg genes as a ratio of the pathway’s total number of genes.

Lastly, the 112 genes of module M1 were used as input for pathway analysis, similar to what was performed for the FIBSig. The aim, again, was to further deduce fibrosis-related inflammatory pathways. Reactome (**Figure 6A**), highlighted immune-related pathways relevant to cytokine signaling, including *IL-4*, *IL-13*, *IFN-α*, *IFN-β*, *IFN-γ*, regulation of signaling, such as that of *IFN-α*, all of which have been already implicated in fibrogenesis. However, we report for the first time the correlation of growth hormone receptors, *CSF3* (*G-CSF*) signaling and pyroptosis with intestinal fibrosis.

Similarly, the investigation of the M1 module with the KEGG database also revealed disease-related pathways, such as those of rheumatoid arthritis, human T-cell leukemia virus 1 infection, *AGE-RACE* signaling pathway in diabetic complications, influenza A, pertussis, human papillomavirus infection, viral myocarditis, and central carbon metabolism in cancer, perhaps due to commonalities in pathogenic mechanisms (**Figure 6B**). In addition, KEGG reported more pathways, seemingly unrelated to fibrogenesis, like those of prolactin, thyroid hormones, hematopoietic cell lineage, c-type lectin receptor signaling and adipocytokines, as well as, cell cycle associated pathways, such as those of p53 and FoxO. Nonetheless, it also highlighted several immune-related pathways that have been associated with both inflammation and fibrosis, such as those of *TNF*, *IL-17*, Th17 differentiation, *JAK-STAT* kinases, and *NOD*-like receptors.

GO:BP analysis revealed some immune- and cell cycle-related pathways, already reported by the previous tools, such as those of signaling and cellular response to *IFN-γ*, epithelial cell proliferation, cell cycle G1/S phase transition and cell number homeostasis (**Figure 6C**). Likewise, GO:MF database highlighted several receptors, molecules and enzymes involved in many of the aforementioned signaling pathways (**Figure 6D**).

## 4 Discussion

In this study, we identified mRNA expression data meeting minimum quality requirements for a wide spectrum of fibrotic diseases, including CD. We then combined the pool of genes differentially expressed in CD with the pool of genes differentially expressed in the rest of the fibrotic diseases. Most of the 241 genes belonging to both pools (FIBSig) were ileum-specific, which is the intestinal segment where fibrosis is most common and most clinically detrimental in CD. Genes were then assembled into pathways and, interestingly, many of them were actually upstream of fibrosis.

We validated the aforementioned *in silico* findings by sequencing mRNA both in the terminal ileum and the colon of CD, UC and controls and cross-sectioned findings with the FIBSig genes ending up to new module of 112 genes (M1). Indeed, localization of most of the M1 genes was again ileal. Starting from this new pool of genes, we employed PPI network analysis to identify 21 pivotal hub genes, that may be of great pharmacologic value. Interestingly, there has been some research on these genes in relation to fibrosis, but no research for any of them relevant to intestinal fibrosis (**Table 4**). Of note, currently there is no effective treatment for fibrotic CD. Lastly, genes of the M1 module were also relevant to a wide range of pathways, not necessarily the ones directly producing extracellular matrix components. This confirms that genes of the M1 module are indeed promising therapeutic targets for preventing fibrosis.

**Table 4:** The role of the 21 hub genes in fibrogenesis.

GENE	ROLE	ROLE IN FIBROSIS
*ATM*	Kinase Regulates cell cycle upon DNA damage Initiates cell cycle arrest either for DNA repairing or apoptosis depending on the extent of DNA damage (54)	Loss of *ATM* attenuates cardiac fibrosis (55)
*FGFR1*	Receptor tyrosine kinase Fibroblast Growth Factor Receptor family (56)	Inhibition of *FGFR1* reduces skin and pulmonary fibrosis (57, 58)
*FBXW7*	F-box protein family Component of the Skp1-Cdc53/Cullin-F-box-protein complex: ubiquitination and protein degradation of various oncoproteins (59)	Overexpression of *FBXW7* reduces hepatic and pulmonary fibrogenesis (60–62)
*ESR1*	Receptor α to estrogen	*ESR1* is overexpressed in pulmonary fibrosis (63) Its activation decreases epithelial-to-mesenchymal transition (64)
*CCND1*	G1 phase cell cycle regulator (65)	Overexpression of *CCND1* has been associated with renal fibrosis (66)
*HIF1A*	Nuclear transcription factor Regulates oxygen homeostasis (67)	*HIF1A* is overexpressed in early stages of renal fibrosis Its inhibition leads to the improvement of fibrosis (68)
*CEBPB*	Leucine zipper transcription factor (69)	*CEBPB* promotes *TGF-β/SMAD3* signalling Its loss of expression reduces pulmonary fibrosis (70, 71)
*NAMPT*	Nicotinamide phosphoribosyltransferase	Overexpression of *NAMPT* attenuates hepatic fibrosis (72) Contradicting results in an animal model of radiation-induced pulmonary fibrosis: the neutralization of *NAMPT* leads to the amelioration of fibrosis (73)
*KYNU*	Kynureninase – an enzyme involved in the biosynthesis of nicotinamide adenosine dinucleotide cofactors from tryptophan (74)	Unknown
*IRF1*	Regulates immune responses Suppresses tumour development (75)	Overexpression of *IRF1* results in renal fibrosis (76)
*SOCS1*	Member of the suppressor of cytokine signalling family JAK/STAT pathway inhibitor (77)	Loss of expression of *SOCS1* aggravates hepatic fibrosis (78)
*ICAM1*	Intercellular adhesion molecule	Inhibition of *ICAM1* reduces cardiac fibrosis (79)
*ETS1*	Transcriptional factor (80)	Inhibition of *ETS1* reduces cardiac fibrosis (81)
*IL7R*	Receptor of IL-7	High expression levels of *IL7R* have been reported in HBV-induced hepatic fibrosis (82)
*MMP1*	Matrix metalloproteinase 1 Degrades ECM components	Inhibition of *MMP1* improves pulmonary fibrosis (83)
*HNF4A*	Transcription factor Regulates several liver-specific genes (84)	Protective against hepatic fibrosis (85)
*CCL2*	Chemokine	*CCL2* has been implicated in cardiac fibrosis (86)
*CASP1*	Caspase involved in apoptosis	*CASP1* has been implicated in hepatic fibrosis (87)
*STAT1*	Transcription factor	Contradictory results: Overexpression inhibits pulmonary fibrosis Upregulation exacerbates pulmonary fibrosis (88, 89)
*SOCS3*	Member of the suppressor of cytokine signalling family *JAK/STAT* pathway inhibitor (77)	Downregulation of *SOCS3* expression results in enhanced diabetic cardiac fibrosis (90)
*HSP90B1*	Heat shock protein with vital role in protein folding and regulation (91)	*HSP90B1* has been found elevated in hepatic fibrosis (92)

Similar to this study, co-expression gene analysis has been recently used to identify functional gene modules, to shed light on specific interactions and to unmask biological processes involved in pathophysiological mechanisms. It has been employed to identify complex mechanisms behind neurological and psychiatric disorders (93–95), immunological and cancer-related responses (96–99), metabolic disorders (100–102) and several biological processes, like fibrosis (103–105) and inflammation (106–108). Tissue-specific (109, 110) and single-cell (111, 112) co-expression studies have provided invaluable insights into the functional interactome of health and disease. The methodological approaches may vary, especially as to the utilization of different bioinformatics tools, but the core concept of co-expression networks, as means to better understand molecular interactions, is invariably valid. In addition, the concept of using networks and network metrics in studying biological processes is nowadays an established practice in biology, medicine, and pharmacology (113–116). Network centrality metrics, such as degree and closeness centralities revealing hub and bottleneck genes, have contributed to further understanding the importance of specific genes in IBD (117, 118) and other disorders (119, 120) and provide novel therapeutic targets (121–123).

Identification of hub genes widened the spectrum of potential therapeutic targets for stenosing CD. We can make informed assumptions and infer the involvement of specific cell types which do play an active role in tissue-specific co-expression networks (124). *ATM* is mainly found in endothelial and epithelial cells (125, 126), *FGFR1* in fibroblasts and epithelial cells (57, 58), *FBXW7* in hepatic stellate mesenchymal, mononuclear and pulmonary epithelial stem cells (60–62), *ESR1* in myofibroblasts and epithelial cells (63, 64), *CCND1* in renal glomerular mesangial and hepatic stellate cells (66, 127), *HIF1A* in renal epithelial cells and cardiac fibroblasts (68, 128), *CEBPB* in hematopoietic and renal epithelial cells (70, 71), *NAMPT* in hepatic stellate and renal glomerular mesangial cells (72, 129), *IRF1* in renal epithelial cells (76), *SOCS1* in hepatocytes and macrophages (78), *SOCS3* in cardiac fibroblasts (90), *ICAM1* in endothelial cells (79), *ETS1* in hepatic stellate and renal epithelial cells (130, 131), *IL7R* in hepatic stellate cells (82), *MMP1* in fibroblasts (83, 132), *HNF4A* in hepatocytes (85), *CCL2* in fibroblasts (86, 133), *CASP1* in hepatic endothelial cells (87) and *STAT1* in macrophages (88, 89). *HSP90B1*, although it has been recently reported to be implicated in fibrosis (92), the specific cell type expressing it, still, remains undetermined.

Pathway databases can identify both broad biological processes and more specific pathways. Different databases provide similar pathway information but annotate them in a completely unique way based on their intended purpose. For this reason, tools like MetaScape (134) and Enrichr (135) provide scientists with a multitude of information from different databases so that they can decide which information better represents their data. As evident in our own analysis too, KEGG is more suitable for discovering common molecular backgrounds among diseases, while GO can provide lower-level information on the cellular mechanisms involved in each pathway. Reactome on the other hand appears to provide a more balanced approach offering several levels of detail which in a case-by-case scenario can either be more or less informative.

For example, in our results IL-13 has been associated with fibrosis in both the FIBSig and the M1 module, and indeed, it is already known to play a pivotal role in various fibrotic diseases, such as SSc, IPF and liver fibrosis (136–138). Another wide group includes pathways of *IFN*- *α*, *β* and *γ* signaling and regulation. IFN-α has anti-fibrotic effects by inhibiting *TGF-β* signaling (139). *IFN -β* and *-γ* have also been recognized as anti-fibrotic cytokines in various organs, such the liver, the lungs, and the kidneys (140–144). Various *TGF-β* signaling pathways also stood out in our study, including the transcriptional activity of *SMAD2/SMAD3/SMAD4* heterodimer, the signaling by *TGF-β* receptor complex, the signaling by *TGF-β* family member and the *RHO GTPase* cycle pathways. *TGF-β* is one of the most well-known pro-fibrotic cytokines and its signal transduction may occur either through the canonical pathway that stratifies *SMADs*, or through non-canonical, such as the *RHO GTPase* pathway (145).

The TNF signaling pathway was also highlighted in both the FIBSig and the M1 module analyzed with the KEGG database. *TNF-α* is a well-known pro-inflammatory cytokine with a role in both inflammation and fibrosis, and many studies have shown that it promotes pulmonary and intestinal fibrosis, through different mechanisms, such as the activation of fibroblasts (146, 147). *IL-17* signaling and differentiation of T cells to Th17 pathways were also highlighted in both the FIBSig and M1 module pathway analysis performed with the KEGG database. Again, both these pathways have been long known for their implication in inflammation and fibrosis. In an animal model of intestinal fibrosis, *IL-17* was found elevated in serum and its neutralization resulted in the amelioration of fibrosis (148). Nonetheless, there is a controversy around *IL-17* as, apart from its fibrogenic role, it also protects from inflammation (149).

We also highlighted pro-fibrotic pathways that had never before been associated with intestinal fibrosis. Specifically, growth hormone receptor signaling was enriched in the M1 fibrotic module. Growth hormones have been associated with liver and pulmonary fibrosis, with most studies concluding that it may have a protective role during fibrogenesis (150–153). *CSF3* probably counteracts fibrosis, as high expression levels have been correlated with reduced ECM deposition in the liver, and its administration in bleomycin-affected mice resulted in the amelioration of pulmonary fibrosis (154, 155). Thus, the inactivation of *CSF3* (*G-CSF*) signaling pathway, may suggest, for the first time, the involvement of this pathway in intestinal fibrosis. Additionally, pyroptosis, a form of programmed cellular death related to inflammation, can be triggered by infectious and non-infectious stimuli (156) and has been linked to fibrosis development in other organs (157–160), but for the first time we demonstrate its implication in intestinal fibrosis. The KEGG database also highlighted *JAK-STAT* signaling, well-known in inflammation, but with no established role in fibrosis yet. *STAT3*, a member of the same signaling pathway, has been shown to promote hepatic fibrosis (161), while *STAT1* was found to counteract *STAT3* and inhibit hepatic fibrosis (162). We also reported *NOD*-like receptor signaling and this is in line with previous studies on hepatic fibrosis showing that its inhibition improves both inflammation and fibrosis (163, 164). *NOD*s in intestinal epithelial cells are intracellular sensors of pathogen-associated molecular patterns and, interestingly, we have shown that gut microbiota differ in stenotic CD (165).

As mentioned, this study capitalizes on previous knowledge from scattered public data and introduces a novel dataset of paired biopsies to identify how fibrosis can be dysregulated in a tissue-specific way during IBD. It also points to therapeutic targets of potential value. Limitations of this study include experimental biases introduced by working with public data obtained under variable conditions in different experimental settings. Further, co-expression analyses rely heavily on sample size, which provides higher statistical power, making it difficult to apply on hard-to-obtain clinical samples

In conclusion, this study, for the first time, highlights novel molecular insights into fibrosis across multiple disorders of different immune pathologies. Composing a new cohort of paired-tissue biopsy samples from the same patients has provided the necessary platform for studying fibrosis in tandem on the terminal ileum and the sigmoid. Enlisting current knowledge along with new data and leveraging state-of-the-art bioinformatics we attempt to go beyond previous works, which focus on known mechanisms of fibrosis (166), and identify new pathways associated with site-specific predisposition towards scarring during IBD. CD strictures in the ileum are far more frequent than in the colon (124). This study provides a molecular level explanation for this dominant phenotype as revealed in the M1 module’s GSEA analysis. Thus, we have shown how co-expression differences can help bring to the foreground localized variations of a ubiquitous phenomenon.

## Data availability statement

The dataset generated by this study containing the RNA-seq data of paired-tissue biopsies can be found under SRA/ENA Project Accession PRJEB56386/ERP141320. The data can also be accessed here: https://www.ebi.ac.uk/ena/browser/view/PRJEB56386.

## Ethics statement

The studies involving human participants were reviewed and approved by Research Ethics Committee University Hospital of Alexandroupolis, Greece (Protocol Number: Q9/D.S37/21.12.2018). The patients/participants provided their written informed consent to participate in this study.

## Author contributions

Conceptualization, ND and EF. Methodology, ND, EF, VV, KA, ID, GB and GK. Validation, ND, EF, VV, VM and GK. Investigation, ND, EF, IK, GT, LK and MS. Resources, GK, GKOK, SV and GB. Writing—original draft preparation, ND, and EF. Writing—review and editing, VP, VM, VV and GK. Visualization, ND and EF. Supervision, GK. Funding acquisition, VP, VM and GK. All authors contributed to the article and approved the submitted version.
